# An Account of the Efficacy of Blood-Letting and Cathartics in the Cure of Palsy, with Observations on That Disease

**Published:** 1810-11

**Authors:** Hunting Sherrill

**Affiliations:** Clinton, Dutchess County, (N. Y.) formerly resident in the New-York Alms-House


*An Account of the efficacy of Blood-Letting and Cathartics in the cure of Palsy, with Observations on that Disease; communicated by Dr.* Hunting Sherrill, *of Clinton, Dutchess County, (N. Y.) formerly resident in the New-York Alms-House, to* Edward Miller, *M. D. Professor of the Practice of Physic in the University of New-York. Dated June 30th, 1810.*

THOSE who have made examinations of the brain, after death, in persons who have been affected with palsy, have ascertained that there is, generally, a compression of the origin of the affected nerves; owing to an extravasation of blood, or an effusion of fluid in some part of the brain. This observation is supported by a number of writers, among whom may be mentioned John Hunter, Townsend, and John Bell. And this appears to be the immediate cause of the nerves, and muscles dependent on them, having lost their power of action. Any case of palsy, where the nerves are injured or compressed, after they emerge from the brain, is not included in this account. In injuries of the head from external violence, where there is a compression of the brain by a depression of the skull, an extravasation of blood, or a collection of matter within the cranium, surgical writers agree that palsy, more or less extensive, is a common symptom, and experience confirms the remark.

Many of the symptoms exhibited by a patient, labouring under hemiplegia, or any considerable extensive attack of

 palsy, palsy, are very similar to those produced by compression of the brain, from local injuries of the head. Both are attended with distress in the head; in both the mental faculties are, generally, somewhat impaired, or are affected, in some degree, with stupor; and both have paralytic affections of the muscles, and they have a striking similarity in the state of the pulse. The practice of surgeons in those cases of local affections is established and uniform. They proceed to remove the compression by applying the trepan, elevating the depressed bone; discharge the blood, which is extravasated within the scull, or the matter which may have collected there; and which commonly affords almost immediate relief of the paralytic symptoms; or if it should not be thought adviseable to use the trepan, (as is frequently the case,) he has recourse to remedies which answer the intention in a more indirect manner, and at the same time prevent inflammation, which are such as those of the depleting class. From the apparent similarity of the effect on the origin of the nerves in the brain produced by those two different causes, we would infer that a similarity of treatment might rationally be adopted in palsies not arising from local causes, to that used to remove an oppressed brain from local injury; with this difference, that while the surgeon, with his instrument, goes directly to the seat of the complaint, and removes the immediate cause by a manual operation, the physician must take a more circuitous rout, and accommodate his remedies to the state of the system, so as to carry off the compression by slow absorption from the brain, to relieve the affected nerves. Conformably with these principles, I made use of some remedies, which it appeared were directed to the true pathology of this disease, in several cases which came under my care in the New-York Alms-house in 1808, the principal of which were blood-letting and cathartics. I was warranted in using blood-letting from its being indicated by this pathology of the disease, and from the states of the pulse noted in the annexed cases; in all which, Dr. Rush would recommend blood-letting, under certain circumstances; (see his *Defence of Blood-letting*,) and from the following observation in Hunter on the Blood, part 2, chap. 1. In palsy, he says, "We ought to bleed at once very largely, "till the patient begins to shew signs of recovery; and to "continue it till he begins to become faint sh." By accommodating this remedy to the morbid actions of the arterial system, without having particular reference to the name of the disease, there appears to be no impropriety in continuing it as long as the state of the pulse and continuance of the symptoms indicate it.

Cathartics

Cathartics are used to promote absorption from other parts of the body in a variety of cases. Dr. Rush informs us, that he has long observed their good effects in palsies, and other cases of congestion in the brain; with this intention, they were used to relieve the oppressed nerves.

The following cases, selected from my case-book, will show the result of this method of treatment. It will be seen that the usual local remedies were used in the most of them, as likewise occasionally galvanism and electricity; but in a number of similar cases, where these were used alone, they appeared to afford very little permanent benefit; and they are probably useful only as auxiliaries to remedies which have a more general effect on the system. It will also be observed that these were principally recent cases. What effect this treatment may have on those of long standing, time and observation alone will determine. From its effects on No. 2, it is more than probable that it may be of service in some of them.

Owing to the obliging disposition of the visiting physicians, Dr. Hosack, and Dr. M'Nevin, these cases came solely under my management.

No. 1.

——— Baxter, aged about 60 years, was attacked with a paralytic affection on the 15th June, 1808, which affected the right arm, hand, and side of the head, attended with considerable headache. It deprived her of the use of the arm and hand; speech was slightly affected; pulse small and firm. She took a cathartic of sub-muriate of mercury, gum aloes, gum gamb. āā gr. viij, and used stimulant friction to the limb. The cathartic produced several evacuations from the bowels. The next day she felt somewhat relieved of the headach and numbness. On the second day the cathartic was repeated. She continued to convalesce. She was electrified once, but so disliked the sensation it produced, that she would not submit to it a second time. The cathartics were repeated once in about three days; the local remedy omitted. In about ten days all the symptoms left her, and she enjoyed the full use of her arm.

No. 2.

James Travis, aged 49 years, was admitted into the Hospital October 28, 1806, with a hemiplegia. When admitted, as I was informed, he had no use whatever of the affected side; was helpless and entirely speechless. By the use of the treatment which had been employed, he had recovered a partial use of his speech; could hobble across the floor indifferently, differently, with assistance; and could give his arm considerable motion. June 20th, 1808; for about six weeks past he has been under a course of treatment recommended by Mr. Rowley, in his treatise on diseases of the eyes, p. 23, consisting principally of mercurials, antimonials, camphor and aloetics, given together or separately in small doses, with vesicatories and stimulants externally; from which, however, he derived no benefit whatever. Several months previous to this, he had been under no particular plan of treatment; pulse small, full and tense; he was bled to sixteen ounces; it nearly produced syncope; pulse better after it; thinks himself better; in a week the bleeding was repeated to 20 oz.; effect as before; he was electrified, and used local stimulants; he walked better and raised his arm higher than ever before since his attack. In another week, has taken 2 or 3 cathartics of sub-muriate of merc. gum gamb. gum aloes, āā gr. 10; they produced several discharges, but were not excessive; bleeding repeated to 24 oz.; slight syncope ensued; gains more use of his limbs. In a week, again; pulse small and tense; bleeding repeated to 24 oz.; syncope ensued; the blood contained yet as great a proportion of crassamentum as at any of the former bleedings; continues friction, with tinct. canth. In two weeks more, has taken cathartics once a week; walks indifferently without a staff; with difficulty, he can raise his arm as high as his head; pulse small, but considerably tense; was bled to 20 oz.; no particular effect ensued; was galvanized daily for two or three weeks, by receiving sparks from Mr. Cruickshank's galvanic trough. It was omitted, principally, for want of time; from the multiplicity and variety of cases that required attention. The depleting plan was continued 5 or 6 weeks longer, but with not so great a degree of steadiness; during which time he was bled 4 or 5 times, and took several cathartics, but did not derive the benefit from it, which he had done, though he gained somewhat. The disease proving obstinate, and the prospect of succeeding being somewhat gloomy, the case was given up; at which time he could walk without a staff, raise his hand to his head with ease, and enjoyed good general health. He remained in about this situation, until about the first of October 1809, during which time he used no particular remedies, as he informs me. Since which, he has gradually lost considerably the use of his limbs again.

No. 3.

D—— M'Lain, aged 28 years, a native of Ireland; was brought to the Alms-house, on a cart, on the 8th July, 1808, completely helpless, from a paralytic affection of the left side.

The

The left arm and leg hung motionless. Had considerable stupor, and a numbness of the whole left side, and of the tongue; he was entirely deprived of his speech; could only make an imperfect guttural sound. By signs and expressions, it was ascertained, that he had little or no pain, except in the head, and some distress in the region of the stomach. The attendants who came with him informed me, that he was suddenly attacked with those symptoms on the 4th of July in the morning, previous to which he had enjoyed good health. His pulse was small, slow, and tense. An emetic was given on the 9th, which discharged a quantity of foul mucous matter from the stomach; emetic was repeated on the 10th and 12th, and rubefacients, with friction, daily; by which his tongue, and the distress at the stomach were somewhat relieved; pulse became more regular. He was now put on a course of cathartics, composed of sub muriate merc. gum gamb. gum aloes, āā 10 gr.; their operation was pretty brisk, but not excessive; they were repeated once in 4 or 5 days; sometimes, however, the gamboge was omitted; at others, the aloes, and sometimes jalap or rhubarb was substituted for one of those. Rubefacients were continued, and were occasionally varied from one article to another of that class. Aug. 1—he had recovered the use of his speech, so as to converse somewhat intelligibly; the use of his leg, so as to move it in any direction, and a trifling power of moving his arm; pulse small and tense; appetite and general health rather improved. Complaint has remained stationary for ten days past. He was now bled to 16 oz. which induced a disposition to syncope; other remedies continued, but the cathartics not so often. After the bleeding, the pulse better; bleeding was repeated, whenever the pulse became preternaturally tense, or so much so as to bear the lancet, which was about once a week. The quantity of blood taken, at each bleeding, was from 12 to 24 oz. He was galvanized through the affected parts. Sept. 1—has been gradually recovering, since pursuing this plan; begins to walk about the ward with a staff; gives the arm considerable motion, and has recovered the use of his speech. The galvanism was continued but about a week, owing in a measure to the reason mentioned in the preceding case, but partly from its not appearing to come within the indication on which the case was treated. The cathartics were repeated one a week, and the bleeding once in two weeks; he was electrified occasionally, and occasionally used friction to the skin. Oct. 1—he has perfectly recovered the use of his leg, and walks about town without a staff, and converses well; has no symptom now remaining but the affection of the arm, which he can raise to a horizontal zontal position, and make some use of it. He enjoys very good general health, and has acquired considerable flesh. Electricity was continued but a short time, being obliged to leave town; the treatment was suspended; soon after which I relinquished my situation in the House, and it has not been resumed since.—He has been under no regular treatment since, as I am informed, and remains in nearly the same situation yet.

No. 4.

A—— B——, about 55 years of age, had long been a resident of the house. Aug. 25; was attacked with a paralytic affection of one of his arms, shoulder, and side of his head; speech considerably affected; attended with headach; pulse slow and firm. He took sub-muriate merc. gum gambog. āā 12 gr.; bathed the arm with vinegar, in which mustard seed had been infused. Next day he was somewhat better. The cathartics were repeated once in 3 or 4 days. In about three weeks from the attack he was discharged from the medical department of the house, cured. To which the following case may be added.

No. 5.

Mrs. —— ——, a lady of this town, aged 27 years, of delicate habit, and slender make; about the middle of December last, was deprived of the use of her right arm and hand; attended with pain in the head, and some vertigo. Local applications were made to the affected limb, of stimulants and frictions; and she took mustard seed in small doses. This kind of treatment was pursued until the 9th February 1810, when I first saw her; at which time the symptoms had increased, so that the headach and vertigo had become very troublesome; had pain and numbness in the right side of her head and face, in the inferior extremity, and in short, in the whole right side. From these symptoms it was concluded, that she was threatened with a more general attack of the complaint. Her pulse was small, slow, and firm. Appetite somewhat impaired; she was bled 6 oz.; took a cathartic, composed of sulphate potash gr. 12, pulv. rhei. gr. 8; submuriate merc. gr. 5, which was repeated every 3 days. An issue was formed on the right side of the base of the os occipitis. She was using powdered mustard seed on the affected arm, which was continued. On the 17th the issue discharged; cathartics operate well; symptoms rather abated; pulse full, small, and tense; was bled to 8 oz. and the other treatment was continued. On the 20th, pain and affection of the lower extremity entirely removed. Vertigo much better; recovered some some use of her arm; general health improved; pulse small, tense, and slow: was bled to 10 oz.; bathe the arm with tinc. canth. daily; repeat the cathartics. March 5th—affection of the face entirely removed; arm much better; pulse tense; was bled to 8 oz.; the blood retains its usual proportion of crassamentum; and this portion, if any thing, had a larger quantity than natural. The cathartics were now taken about once a week; omitted the tinc. canth. and merely rubbed the limb. She continued to convalesce. By the 17th March, had recovered the use of her arm, except the wrist and hand; when she awoke in the night, and found herself entirely relieved of all the paralytic symptoms, and enjoyed the full use of her arm, except a slight weakness and some headach only remaining; since which, she has fully recovered.

